# In-Office Tympanostomy Tube Placement Using Iontophoresis and Automated Tube Delivery Systems

**DOI:** 10.1177/2473974X20903125

**Published:** 2020-02-24

**Authors:** David M. Yen, Michael T. Murray, Robert Puchalski, Andrew R. Gould, John Ansley, Randall A. Ow, Jonathan R. Moss, Laura J. England, Charles A. Syms

**Affiliations:** 1 Specialty Physician Associates, Bethlehem, Pennsylvania, USA; 2 Camino Ear, Nose & Throat Clinic, San Jose, California, USA; 3 South Carolina ENT Allergy & Sleep Medicine, Columbia, South Carolina, USA; 4 Advanced ENT & Allergy, Louisville, Kentucky, USA; 5 Carolina Ear Nose & Throat Clinic, Orangeburg, South Carolina, USA; 6 Sacramento Ear, Nose and Throat, Roseville, California, USA; 7 Charlotte Eye Ear Nose & Throat Associates, Matthews, North Carolina, USA; 8 Tusker Medical, Menlo Park, California, USA; 9 Ear Medical Group, San Antonio, Texas, USA

**Keywords:** iontophoresis, tympanostomy tube, myringotomy, local anesthesia, office surgery, pediatric

## Abstract

**Objectives:**

(1) To evaluate safety, tolerability, and technical success of lidocaine iontophoresis and a tympanostomy tube placement system for adults in an office setting and (2) to meet regulatory evidence requirements for new drugs and devices.

**Study Design:**

Prospective, multicenter, single arm.

**Setting:**

Patients were recruited in 8 community-based practices in the United States between June and September 2017.

**Subjects and Methods:**

This study evaluated tympanic membrane anesthesia and tube placement in 30 adults. Anesthesia was achieved via iontophoresis of a lidocaine/epinephrine solution. Tube placement was conducted using an integrated myringotomy and tube delivery system. Tolerability of tube placement was measured using a patient-reported visual analog scale from 0 mm (*no pain*) to 100 mm (*worst possible pain*). Mean pain score was compared to a performance goal of 45 mm, where statistical superiority represents mild pain or less. Technical success and safety through 3 weeks postprocedure were evaluated.

**Results:**

Twenty-nine (29/30, 96.7%) patients had tube(s) successfully placed in all indicated ears. One patient demonstrated inadequate tympanic membrane anesthesia, and no tube placement was attempted. The mean (SD) pain score of 9.4 (15.7) mm was statistically superior to the performance goal. There were no serious adverse events. Seven nonserious events were related to device, procedure, or drug: inadequate anesthesia (1), vertigo (1), and dizziness (1) at the time of procedure and ear discomfort (1), tube occlusion (2), and medial tube migration (1) postprocedure.

**Conclusion:**

Lidocaine iontophoresis provides acceptable tympanic membrane anesthesia for safe, tolerable, and successful in-office tube placement using an integrated myringotomy and tube delivery system.

In adults and older children, tympanostomy tubes can be placed in an office setting using a variety of local anesthesia options. Phenol (carbolic acid) is used to cause a partial-thickness chemical burn, leading to localized anesthesia of the tympanic membrane (TM).^1^ Other local anesthetics, such as EMLA cream (eutectic mixture of local anesthetics, lidocaine 2.5%, prilocaine 2.5%), lidocaine injections, Bonain’s solution (cocaine hydrochloride, menthol, phenol), and tetracaine injections, are used less frequently.^2-4^ None of the local anesthetics employed in adults are regularly used with young children due to discomfort, poor reliability, or lengthy onset incompatible with pediatric use, and none have US Food and Drug Administration (FDA) approval for this use.

Tympanostomy tube procedures comprise >20% of surgical procedures performed in children younger than 15 years in the United States, making it one of the most common surgeries performed in children.^5^ By age 3, almost 7% of children will have tympanostomy tubes.^6^ Nearly all tympanostomy procedures in children are performed in the operating room (OR) under general anesthesia.

The Tula Iontophoresis (IPS) and Tube Delivery Systems (TDS) (Tusker Medical, Menlo Park, California) were developed with the intent to enable safe and reliable placement of tympanostomy tubes in an office setting with localized administration of anesthetics to the TM. For the pediatric population, the technologies provide the capability to avoid risks and side effects of general anesthesia, reduce patient and parental anxiety associated with OR-based procedures, and move tube placement procedures to a lower cost setting.

The purpose of this investigation is to evaluate safety, tolerability, and technical success of the Tula system in adults, as required by the FDA, to confirm system suitability prior to initiating pediatric investigation. The study was designed with input from physician advisers to meet FDA requirements and was industry sponsored. The adult population is relevant to interpretation of pediatric risk because TM size and mass are the same for children and adults; therefore, the anesthesia effect is expected to be similar.^7^ In addition, adult data are relevant to understanding systemic exposure because lidocaine and epinephrine pharmacokinetics are similar for children >6 months of age.^8,9^

## Methods

### Study Design

The ADEPT study (ClinicalTrials.gov NCT03197558) was a prospective multicenter, single-arm study evaluating safety, tolerability, and technical success of tympanostomy tube placement using the TDS in adults following local anesthesia using the IPS in an office setting. Patients were recruited in 8 community-based practices between June and September 2017, with each surgeon-author performing study tube placement procedures. The study was conducted in accordance with Good Clinical Practices and ethical principles of the Declaration of Helsinki. The study protocol was approved by the Western Institutional Review Board for each participating center, and informed consent was obtained from all patients.

### Patient Population

Inclusion criteria were adults aged ≥18 years indicated for unilateral or bilateral tympanostomy tube insertion per the American Academy of Otolaryngology–Head and Neck Surgery (AAO-HNS) Clinical Practice Guideline or for barotrauma or eustachian tube dysfunction per AAO-HNS Clinical Indicators.^2,10^ Exclusion criteria consisted of medical history that would affect drug delivery or patient safety, including known sensitivity or insensitivity to local anesthetics; significant atrophic, retracted, bimeric, or perforated TMs; otitis externa; damaged or denuded skin in the external auditory canal; anatomy that precluded sufficient visualization of the TM or that necessitated tube placement in the posterior TM; electrically sensitive support systems (eg, pacemakers, defibrillators); and pregnant or lactating women. Patients were excluded if cerumen removal prior to iontophoresis resulted in a significant amount of cleaning with potential for irritation.

### Iontophoresis System

**Figure 1.**
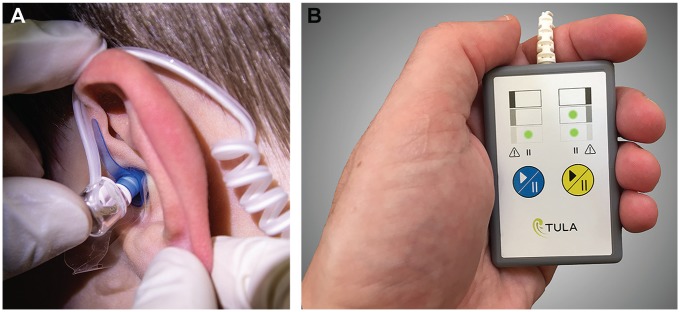
(A) Iontophoresis System Earset. (B) Iontophoresis System Control Unit.

Local anesthesia of the TM was accomplished with the IPS (earset, **Figure 1A**; control unit, **Figure 1B**) using iontophoresis of 2% lidocaine and 1:100,000 epinephrine (Hospira, Lake Forest, Illinois). The IPS is a portable, battery-powered, microprocessor-controlled, direct-current generator that provides a low-level electrical current (0.8 mA) to the ionic drug solution and permits simultaneous bilateral anesthesia during the iontophoresis process (approximately 10-15 minutes). Specialized earplugs maintain the drug solution in contact with the TM, permitting patient mobility. No anxiolytics or sedatives were used. Iontophoresis duration from initiation to completion of the current delivery cycle was recorded.

### Tube Delivery System

**Figure 2.**
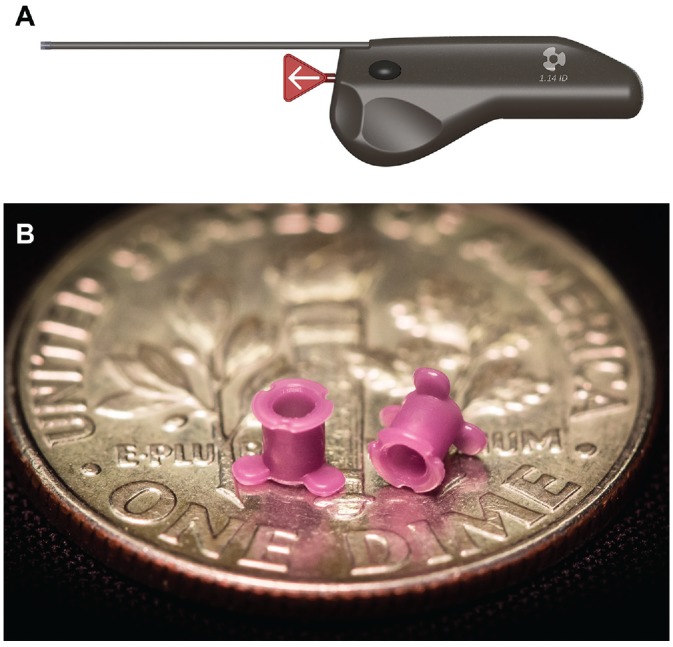
(A) Tube Delivery System. (B) Tube Delivery System tube.

Tube placement was performed using the TDS (**Figure 2A**), a mechanical device that automates rapid myringotomy and tube placement. With the tip of the TDS held apposed to the anesthetized TM, an actuation button executes precise myringotomy and tube placement in <0.5 seconds, with the cutting blade exposed for <0.25 seconds. The silicone tube has an internal diameter of 1.14 mm (**Figure 2B**).

### Local Anesthesia and Tube Placement Procedure

Baseline measures included otoscopy, tympanometry, and audiometry up to 28 days preprocedure.

Patients underwent iontophoresis in each ear that required tube insertion, with bilateral patients receiving simultaneous bilateral iontophoresis. Anesthetic solution was warmed to minimize vestibular caloric effects. Following iontophoresis, earplugs were removed and anesthetic solution was cleared from the ear canal by wicking or gravity. Once anesthesia was confirmed by patient reaction to lightly touching the TM using a dull otologic instrument, the TDS was used to deliver the tube. If middle ear effusion was present, it was gently suctioned through the tube following tube placement at the investigator’s discretion. Otic drops were administered based upon each patient’s condition.

To evaluate potential systemic effects of anesthetic, vital sign measurements (pulse, blood pressure, respiratory rate, and blood oxygen saturation) were taken prior to the procedure, after iontophoresis, after tube placement, and 30 and 60 minutes postprocedure.

One follow-up visit occurred at 3 weeks (±7 days) postprocedure and included otoscopy, audiometry, and tympanometry. Tube presence and patency were assessed. Adverse events at procedure through follow-up were recorded.

### Outcome Measures

Anesthesia effectiveness and tolerability were evaluated via patient-reported pain ratings using a visual analog scale (VAS). The VAS is a validated instrument for assessing pain consisting of a 100-mm line, where the ends of the line represent the extreme limits of pain (0 mm = *no pain* and 100 mm = *worst possible pain*).^11^ Patients reported pain intensity by making a mark along the line, which was measured to generate a numeric score. A standardized instructional script was used to ensure consistent interpretation of the pain rating task. Pain scores were collected prior to the procedure and after TM anesthesia assessment, tube placement, and suction. After the procedure, patients were asked, “Did the local anesthetic provide adequate pain relief for the tube placement procedure?”

Technical success was defined as the proportion of patients receiving TDS-inserted tubes in all indicated ears. Bilateral patients must have had tubes successfully placed in both ears to count as a technical success.

Air conduction pure-tone average hearing threshold changes (>15 dB compared to baseline), vital sign changes (≥30% compared to baseline), or clinically significant changes were considered adverse events.

### Sample Size and Statistical Analysis

The statistical analysis plan was defined prior to enrollment. Tube placement tolerability was assessed using a patient-level VAS score. If both ears were treated, the highest score of the 2 ears served as the patient-level score. The statistical test for the tube placement tolerability end point compared mean VAS score to a performance goal of 45 mm, where pain scores below 45 mm have been shown to correspond to mild pain.^12^ The end point was demonstrated if the upper confidence bound of a 95% nonparametric bootstrap confidence interval on the mean score was below 45 mm.

Using bootstrap simulation, a 30-patient sample was estimated to provide >95% power at a 1-sided 2.5% significance level.

Additional analyses for technical success, anesthesia effectiveness, suction tolerability, tube patency, and retention were summarized using descriptive statistics. Patient-reported anesthesia effectiveness and suction tolerability were determined from the VAS score reported by ear.

SAS system (v9.2 or later; SAS Institute, Cary, North Carolina) or R version 3.3.2 (R Foundation for Statistical Computing, Vienna, Austria) were used to perform all data analyses.

## Results

### Patient Population and Procedural Data

Eight centers enrolled 30 adults ages 21 to 83 years (mean [SD] 54.9 [15.7] years), with 57% female. The 30 treated patients included 38 ears undergoing iontophoresis with a mean (SD) duration of 10.5 (1.4) minutes. Iontophoresis was successfully completed for all ears. Two patients experienced 2 iontophoresis interruptions due to earplug leak, and 1 patient required earset replacement due to improper sizing. For 3 patients, the electrical current level was reduced to effectively address discomfort during iontophoresis.

Iontophoresis was successful in providing TM anesthesia in 29 of 30 subjects. Inadequate anesthesia in 1 unilateral patient was determined by the physician based on the patient’s verbal and physical response to touching the TM. Tube placement was not attempted. Subsequent discussion revealed the patient was insensitive to dental anesthetics and should have been excluded from participation because history of insensitivity to local anesthetics was a defined exclusion.

The 29 remaining patients had a mean (SD) VAS score after TM touch of 2.9 (7.6) mm. These 29 patients had 37 ears undergo tube placement. There were 21 of 29 (72.4%) unilateral and 8 of 29 (27.6%) bilateral patients. All 29 of 29 patients and 37 of 37 ears had the tube successfully placed across the TM.

Following tube placement, suction to remove middle ear effusion was performed in 7 of 37 (18.9%) ears (7 patients), and mean (SD) VAS score for the suction procedure was 4.1 (8.8) mm (**Table 1**).

**Table 1.** Patient-Reported VAS Pain Scores (in mm, on a 0-mm to 100-mm Scale).

	VAS Pain Score, mm N = 29 Patients (37 Ears)	
Characteristic	Mean (SD) (No.)	Median
Outcomes by patient		
Preprocedure	1.3 (2.7) (29)	0.0
After tube placement	9.4 (15.7) (29)	3.0
Outcomes by ear		
Preprocedure	1.1 (2.4) (37)	0.0
TM touch anesthesia assessment	2.4 (6.8) (37)	0.0
Postsuction	4.1 (8.8) (7)	1.0

Abbreviations: TM, tympanic membrane; VAS, visual analog scale.

### Tube Placement Tolerability

Mean (SD) tube placement VAS score was 9.4 (15.7) mm. Tube placement tolerability by patient and by ear is summarized in **Table 1**. Mean subject-level score was compared to a performance goal of 45 mm at a 2.5% significance level. The upper confidence limit was 14.35 mm, below the performance goal, meeting the primary end point.

All 29 of 29 patients who underwent tube placement responded they felt the local anesthetic provided adequate pain relief for the tube placement procedure.

### Tube Patency and Tube Retention

All 29 of 29 patients completed the 3-week follow-up visit. The tube was present in the correct position across the TM for 28 of 29 patients (96.6%) and 36 of 37 ears (97.3%). The tube migrated medially in 1 patient who reported no symptoms. There were 26 of 28 patients (92.9%) and 34 of 36 ears (94.4%) with patent tubes at the 3-week visit.

### Safety

There were no serious adverse events. Seven nonserious events were related to device, procedure, or drug: inadequate anesthesia (1), vertigo (1), and dizziness (1) at time of procedure; ear discomfort (1) 1 day postprocedure; and tube occlusion (2) and medial tube migration (1) at 3-week follow-up. There were no adverse events related to hearing or vital signs.

The patient who experienced medial tube migration returned at the 3-week visit, and while middle ear effusion had resolved, the tube was visualized in the middle ear behind the intact, healed TM. Hearing loss remained mild, low-frequency mixed with modest improvement in the conductive component. Tympanometry improved from type B to C. The patient returned 2 months postprocedure showing further improvement in hearing, and tympanogram changed to normal (type A). Per investigator standard of care, no intervention was performed on the asymptomatic medialized tube. This patient exited the study 59 days postprocedure into the investigator’s ongoing care with the tube medialized but asymptomatic and with no clinical sequelae.

## Discussion

The IPS, TDS, and iontophoretic drug used in this study did not have regulatory approval at the time of this evaluation. The FDA process requires the technology manufacturer to have approval from the FDA on the clinical protocol, the results of which will be reviewed by the FDA to permit marketing. The manufacturer provided the technology via an FDA-approved Investigational Device Exemption (IDE), financially supported the study, and designed the study in accordance with regulatory requirements and physician adviser input. Bias is a potential limitation of the study and was managed by using an objective, binary end point (technical success). Study data were 100% verified against medical records, and an independent nonenrolling ear, nose, and throat surgeon and an audiologist adjudicated all adverse events.

Iontophoresis for TM anesthesia has been described as early as the 1970s using custom or marketed iontophoresis technologies (Medtronic Xomed Ionesthetizer, Jacksonville, FL; Life-Tech Otophor, Houston, TX).^13-26^ Limitations of these devices included extended procedure duration, inability to treat both ears simultaneously, and a requirement for the patient to lie on one side. The devices used a bare anode that could contact the ear canal, potentially resulting in discomfort or burn. These limitations made the products challenging to use in young children. The IPS described in this report incorporates features to address these limitations for translation into pediatric application.

Technology enabling in-office tube placement in young children offers several advantages. Perioperative risks associated with general anesthesia required for OR tube placement can be avoided. Although uncommon, adverse effects of general anesthesia during tube procedures can be severe, including laryngospasm (0.9%), severe airway obstruction (1.4%), blood oxygen desaturation (0.4%), dysrhythmia (1.8%), and postoperative vomiting requiring treatment (0.4%).^27,28^ Up to 57% of children undergoing general anesthesia with sevoflurane for tube placement show emergence delirium, defined as ≥3 minutes of thrashing requiring restraint.^29^

A growing body of literature suggests a linkage between general anesthesia and neurodevelopmental impact, although it is difficult to differentiate effects of anesthetic compared to the underlying medical condition. One study showed that children who received general anesthesia before age 3 were 1.9 times as likely to show language disability and 1.7 times as likely to show cognitive disability, even after a single anesthetic exposure.^30^ In another study, children with multiple exposures to general anesthesia before age 4 had increased risk of developing learning disabilities compared to those with a single exposure or none.^31^ A recently published study, however, indicates that a single brief general anesthetic exposure in early infancy does not alter neurodevelopmental outcome at age 5 compared with awake-regional anesthesia.^32^ These findings are relevant for children undergoing tube placement because the most common age for tympanostomy procedures occurs between the neurodevelopmentally important ages of 6 and 36 months, and approximately 20% of children will undergo a second tube placement procedure.^33,34^

One recent report describes pediatric tube placement in a surgery center using oral midazolam, relatively high levels of nitrous oxide (50%-70%, moderate or “conscious” sedation), and phenol application to the TM. Even with conscious sedation, 11.7% of children converted to general anesthesia with sevoflurane to complete tube placement.^35^ Since nitrous oxide is known to be neurotoxic in juvenile animal models and the procedure requires an anesthesia professional and OR or sedation suite setting, such an alternative does not appear to afford significant benefits relative to traditional tube placement using general anesthesia.^36,37^

The Tula IPS and TDS have been reported previously using prior generations of the technologies and a bedside mixture of lidocaine, epinephrine, and sodium bicarbonate for local anesthesia of the TM.^35,38,39^ The IPS enabled myringotomy or myringotomy with tube placement procedures, with a high rate of technical success (89%-90%) in children as young as 8 months with good tolerability and safety outcomes and without use of a papoose, premedication, anxiolysis, or sedation. Analysis of video recordings of the procedures using the validated FLACC (Face, Legs, Activity, Cry, Consolability) scale indicated low distress.^40,41^ Favorable ratings for overall satisfaction with the in-office procedure were obtained from 96.9% (63/65) of parents.^39^

The current study was intended to evaluate newer generations of IPS and TDS technologies and a preformulated iontophoretic lidocaine and epinephrine drug solution in adults prior to further pediatric investigation.

In the current study, all patients achieved successful anesthesia for tube placement except 1 patient who subsequently revealed an insensitivity to local anesthetics. The prevalence of anesthetic insensitivity is unknown, and for children, a history of dental anesthetic experience may not be available. A larger pediatric study is ongoing and will enable an estimate of anesthesia success in children. All 29 adults with TM anesthesia in the current study had successful TDS tube placement.

Patient-reported VAS scores following TDS tube placement showed low/mild discomfort. All patients indicated the anesthetic provided adequate pain relief for the tube placement procedure.

Tube patency and retention rates at 3 weeks postprocedure were consistent with published reports.^42,43^ Occlusion is an anticipated event for any tube and has been reported to occur in 7% to 37% of tubes.^42^ Tube retention rate in the present study (97.3% of ears) was consistent with the retention rate reported by Song et al,^43^ with 97.7% tubes retained within 1 month postoperatively. One ear without a retained tube in this study resulted from medial tube migration, reported in the literature at rates of 0.5% to 1.1%.^42,44^ Others have noted it is likely that such events are underreported because medialized tubes are not always visible under otoscopy.^44-46^ The cause of the medial tube migration is uncertain. The patient’s history of middle ear negative pressure may have played a role in the event.

The patient with vertigo had symptoms consistent with lidocaine in the middle ear. This patient had bilateral myringotomy 12 days prior to the study procedure. Although otoscopy and tympanometry indicated the TM was intact, crusts were observed on the healing TM, and ingress of the drug solution through the healing wound is possible. The symptom and its duration (several hours) are consistent with lidocaine in the middle ear, based on publications of middle ear lidocaine injection to treat severe tinnitus.^47,48^ Exclusion for recent ear conditions or interventions should be included in future studies to mitigate recurrence of this event.

The strengths of this study include its design as a prospective, multicenter trial with a predefined analysis plan and powered performance goal based on a clinically relevant metric (tolerability in the mild pain range or better). The multicenter nature of the study supports generalizability of the results.

A constraint of this study is evaluation in adults limiting generalization of performance to a pediatric population. A second limitation of this study is lack of a control group to compare safety and tolerability to standard tube placement in adults. The goal of the study was not to compare the investigational system to existing options but rather to demonstrate safety and efficacy prior to use of the investigational system for pediatric subjects.

This study demonstrated adequate in-office TM anesthesia for TDS tube placement using lidocaine iontophoresis for adults. The safety evaluation demonstrated an absence of serious adverse events and a low frequency of nonserious adverse events. Based on tube placement tolerability, anesthesia effectiveness, technical success, tube retention and patency rates, and safety reported in this study, TM anesthesia via lidocaine/epinephrine iontophoresis adequately provides TM anesthesia for safe and tolerable in-office tube placement.

## Author Contributions

**David M. Yen**, review of study protocol, acquisition of data, review of aggregated data, drafting and revision of manuscript, final approval of manuscript, accountable for the work; **Michael T. Murray**, acquisition of data, review of aggregated data, manuscript review and revision, final approval of manuscript, accountable for the work; **Robert Puchalski**, acquisition of data, review of aggregated data, manuscript review and revision, final approval of manuscript, accountable for the work; **Andrew R. Gould**, acquisition of data, review of aggregated data, manuscript review and revision, final approval of manuscript, accountable for the work; **John Ansley**, acquisition of data, review of aggregated data, manuscript review and revision, final approval of manuscript, accountable for the work; **Randall A. Ow**, acquisition of data, review of aggregated data, manuscript review and revision, final approval of manuscript, accountable for the work; **Jonathan R. Moss**, acquisition of data, review of aggregated data, manuscript review and revision, final approval of manuscript, accountable for the work; **Laura J. England**, design of study protocol, data analysis, drafting manuscript, final approval of manuscript, accountable for the work; **Charles A. Syms III**, design and review of study protocol, acquisition of data, review of aggregated data, manuscript review and revision, final approval of manuscript, accountable for the work.

## Disclosures

**Competing interests:** David M. Yen, consultant: Tusker Medical; consultant, speaker: Arrinex; consultant, speaker: Intersect ENT; consultant: OmniGuide; consultant, shareholder: SurgeonCheck; consultant, speaker: Stryker. Laura J. England, employee: Tusker Medical. Andrew R. Gould, research funding: Tusker Medical. Michael T. Murray, consultant: Tusker Medical. Randall A. Ow, consultant: Tusker Medical; consultant: Arrinex; speaker: Optinose; consultant, speaker: Spirox. Charles A. Syms, advisory board, shareholder: Tusker Medical; advisory board, shareholder: Earlens, shareholder: Arrinex.

**Sponsorships:** Tusker Medical: study protocol design, provided devices, data analysis, drafting manuscript, logistical support, approval of manuscript.

**Funding source:** Tusker Medical: study protocol design, provided devices, data analysis, drafting manuscript, logistical support, approval of manuscript.
